# Spheroid-Exosome-Based Bioprinting Technology in Regenerative Medicine

**DOI:** 10.3390/jfb15110345

**Published:** 2024-11-14

**Authors:** Hwa-Yong Lee, Jin Woo Lee

**Affiliations:** 1 Division of Science Education, Kangwon National University, Chuncheon 24341, Republic of Korea; leehy@kangwon.ac.kr; 2 Department of Molecular Medicine, College of Medicine, Gachon University, Incheon 21999, Republic of Korea

**Keywords:** bioprinting, exosome, spheroid, regenerative medicine, tissue engineering

## Abstract

Since the discovery that exosomes can exchange genes, their potential use as tools for tissue regeneration, disease diagnosis, and therapeutic applications has drawn significant attention. Emerging three-dimensional (3D) printing technologies, such as bioprinting, which allows the printing of cells, proteins, DNA, and other biological materials, have demonstrated the potential to create complex body tissues or personalized 3D models. The use of 3D spheroids in bioprinting facilitates volumetric tissue reconstruction and accelerates tissue regeneration via exosome secretion. In this review, we discussed a convergence approach between two promising technologies for bioprinting and exosomes in regenerative medicine. Among the various 3D cell culture methods used for exosome production, we focused on spheroids, which are suitable for mass production by bioprinting. We then summarized the research results on cases of bioprinting applications using the spheroids and exosomes produced. If a large number of spheroids can be supplied through bioprinting, the spheroid-exosome-based bioprinting technology will provide new possibilities for application in tissue regeneration, disease diagnosis, and treatment.

## 1. Introduction

Stem cells, which can differentiate into various cell types in the body, can be easily stored, expanded, and transplanted into other people [1]. Because of these advantages, stem cell-based therapies have been actively developed. However, the therapeutic effects of stem cell therapies are not due to the direct replacement of damaged cells or tissues by stem cells but are primarily the result of paracrine effects from various factors secreted by stem cells [2]. Moreover, stem cell therapies have limitations in their application owing to the potential risk of tumor formation [3].

Recent research on stem cell therapies has focused more on utilizing substances secreted by stem cells than on the direct administration of the stem cells themselves. Stem cell-secreted extracellular vesicles (EVs) containing nucleic acids, proteins, and lipids, can mediate tissue regeneration by delivering these components to the local microenvironment of damaged cells or tissues [4,5]. Initially, EVs secreted by most eukaryotic and prokaryotic cells were considered to be waste products. However, since the mid-1990s, when EVs were discovered to play a role in immune responses, they have demonstrated clinical potential as drug-delivery vehicles, therapeutics, and diagnostic tools [6,7,8]. Lipid-bound EVs secreted by cells can be classified into three subtypes: exosomes, micro-vesicles, and apoptotic bodies, each with unique characteristics based on their biosynthesis, release pathways, size, content, and function [9]. Among these, exosomes, which are lipid bilayer vesicles of 30–150 nm in size that originate from the endosomal membrane, are of particular interest [8,10,11,12,13]. Proteins, lipids, and nucleic acids within exosomes vary depending on the state and environment of the secreting cells. The key proteins include annexins, tetraspanins, heat shock proteins, and flotillins, all of which play crucial roles in exosome function and reflect the physiological state of cells [14]. The lipid components of exosomes include cholesterol, sphingolipids, phosphoglycerides, and ceramides, and their glycan-rich structures are key to their signaling and structural functions [15]. Exosomes also contain microRNAs (miRNAs) involved in secretion, hematopoiesis, and angiogenesis, making them attractive for use in disease diagnostics, drug delivery, and therapeutic modulation [16,17,18]. Exosomes have great potential in regenerative medicine because they are involved in physiological processes through intercellular communication and reflect the state of the cells from which they originate. Current research on exosomes focuses on their efficacy in three main applications: therapeutics, diagnostics, and drug delivery vehicles [19,20,21].

Exosomes have uniquely high targeting capabilities, and numerous studies have demonstrated both autocrine and paracrine effects. For example, cancer cells interact via exosome-mediated communication to facilitate metastasis. Exosomes can also be engineered to modify their targeting behavior by attaching specific types of antibodies [22]. As exosomes are less immunogenic than their originating cells, they offer a promising opportunity to improve current therapeutic systems [21]. One example is the use of exosomes in the treatment of cardiovascular diseases. Given their ability to carry proteins and messenger RNAs (mRNAs) between cells, exosomes can promote angiogenesis, enhance coagulation, exert anti-inflammatory effects, and regulate cardiomyocytes and endothelial cells, leading to the formation of new blood vessels and restoration of the oxygen supply to hypoxic regions [11]. Exosomes derived from mesenchymal stem cells (MSCs) have been studied for the treatment of various diseases, including cancer, neurodegenerative diseases, and immune disorders [22,23,24]. For instance, exosomes from adipose-derived MSCs have been shown to protect against myocardial damage [25], whereas exosomes from bone marrow-derived MSCs (BMSCs) promote neural regeneration [26]. Clinical trials using MSC-derived exosomes are currently underway, and various therapeutic strategies are being developed [27].

Because exosomes reflect the cellular origin and condition, they have potential as biomarkers for diseases. Exosomes from tumors, stromal cells, and immune cells are involved in tumor growth, signaling, and treatment resistance, making them useful for cancer diagnosis and monitoring immunotherapy, as they are found in various bodily fluids such as plasma, saliva, and urine [28]. The up-regulation of miRNA and proteins in cancer cell-derived exosomes can aid in the early detection and prediction of treatment responses in various malignancies, including ovarian, breast, cervical, gastric, pancreatic, and lung cancers [29,30]. Exosomes are also being explored as biomarkers of neurodegenerative diseases, diabetes, and cardiovascular diseases [31,32,33,34,35,36]. They have been identified in amyloid-beta plaques in Alzheimer’s disease [31], and miRNAs associated with multiple sclerosis [34], type 1 diabetes, and insulin resistance have been observed in exosomes [35,36].

Compared to synthetic drug carriers, exosomes derived from patients’ cells have high biocompatibility and low toxicity, making them ideal for drug delivery. Exosomes can cross the blood-brain barrier and can be modified on their surface to enhance tissue specificity [37,38]. Studies have shown that using exosomes to deliver anticancer drugs, such as doxorubicin (Dox) and paclitaxel (PTX) reduces cytotoxicity and increases therapeutic efficacy. For instance, Dox-loaded exosomes exhibited lower cytotoxicity and easier absorption, while PTX-loaded exosomes demonstrated potent anticancer effects in a lung metastasis model [39,40]. Moreover, exosomes have been shown to suppress tumors and protect neurons by delivering gemcitabine for pancreatic cancer treatment or dopamine for Parkinson’s disease [41,42]. These examples highlight the potential of exosomes as drug-delivery systems.

However, challenges such as the lack of standardization in exosome isolation and purification techniques, low yield, and variability in the drug delivery potential of different exosomes remain unaddressed [31,43]. The presence of protein mixtures during the isolation process may affect the recovery yield and biological activity of the exosomes. Further improvements and standardization of exosome isolation techniques are necessary [44].

## 2. Production of Exosomes

Traditionally, cells have been cultured in two-dimensional (2D) monolayers on flat glass or plastic surfaces because of their simplicity and convenience [45]. However, 2D cell cultures limit cell–cell and cell–extracellular matrix (ECM) interactions, thereby restricting cellular responsiveness. Consequently, toxicity testing of materials and substances in 2D cultures cannot fully predict the in vivo effects [46,47]. In addition, when cells are cultured in 2D flasks, the surface area of the culture vessel is proportional to cell growth and the number of exosomes produced, making large-scale EV production inefficient [48]. Therefore, 3D culture methods have been employed to grow cells in a more natural, in vivo-like state, with the aim of producing exosomes while minimizing changes in cell tissues [49,50].

**Figure 1:**
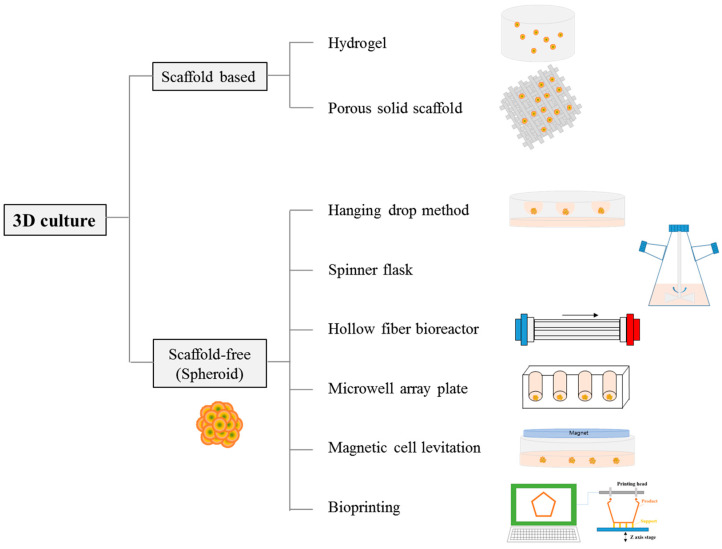
3D cell culture methods for exosome production.

Three-dimensional cell culture methods can be broadly categorized into scaffold-based, natural or synthetic polymer-based, and scaffold-free methods (Figure 1). Scaffold-based methods can be further divided into approaches that use hydrogels and those that use porous solid scaffolds [51]. Hydrogels are created by crosslinking basic materials such as collagen, fibrin, hyaluronic acid, or agarose in a loose, swellable form, encapsulating the cells within the hydrogel for cell growth [52]. Furthermore, hydrogels can be designed to enhance specific cell growth and functions by adding artificial ECM protein environments or encouraging cells to migrate inward from the gel surface [53,54,55]. However, when cells are seeded on porous solid scaffolds, the scaffold provides a 3D space for cell attachment and protection from external forces. The interconnected pores allow for natural cell–cell interactions, forming structures similar to natural 3D tissues as the cells grow. Because scaffold structure control and reproducibility are achievable, scaffold-based 3D cultures can create consistent tissue models in vitro [51]. Yu et al. developed a self-assembling collagen hydrogel and demonstrated that the exosome production yield in their gel was 2.5 times higher, with the protein content increasing by 2.9 times [56].

Scaffold-free methods rely on the natural adhesion between cells to form spheroids without attachment to a scaffold. Several scaffold-free spheroid formation techniques have been reported [47,49]. The hanging drop method is a 3D culture technique that uses a mechanism to collect cells at the center of a droplet, allowing them to spontaneously aggregate [57,58]. The advantage of this method is that spheroids are formed without requiring a support structure. Additionally, the hanging drop method is relatively simple, easy to perform, and cost-effective compared with other methods. Kim et al. produced mesenchymal stem cell (MSC)-derived spheroids and found that exosome production from the spheroids was twice as high as that from 2D cultures [59]. Giusti et al. used the hanging drop method to create 3D spheroids of cancer cells that mimicked in vivo tumor characteristics [60]. However, producing only one spheroid per droplet is time-consuming, and there is a size limitation for spheroids [61]. The limited production yield of the hanging drop method is directly related to difficulties in extracting exosomes and the inability to change the culture medium [62].

A spinner flask is a 3D cell culture system that gently stirs a cell suspension in a medium, causing the cells to aggregate into spheroids [63]. The spinner flask has two side caps that uniformly regulate the oxygen concentration. The ability to produce large quantities of spheroids via mass culture is a key advantage of this system. Exosomes isolated from MSCs cultured in a spinner flask delivered small interfering RNAs (siRNAs) to neurons more effectively than those derived from 2D MSC cultures [64]. However, shear stress caused by rotation can damage cultured cells [65], and the size of the spheroids produced is often inconsistent, requiring careful control of the stirring speed [66,67]. Furthermore, a serum is required for cell aggregation in the spinner flask; however, because cells must be cultured in a serum-free medium for exosome isolation, this can be a drawback for exosome production.

A hollow-fiber bioreactor is a 3D cell culture system in which multiple hollow, semi-permeable fiber membranes are placed within flowing media. In 3D cultures, cells are seeded either inside or outside the fibers, which are approximately 200 µm in diameter. Gas and nutrient exchanges occur through semi-permeable membranes. The medium typically flows in one direction to remove waste from the culture [68]. The closed-circuit configuration of the hollow-fiber bioreactor allows for the automatic infusion of nutrients and oxygen through sealed tubes, thereby reducing labor. By connecting multiple hollow fibers to a single bioreactor, large-scale exosome production is possible, making it suitable for automated exosome mass production. Recent studies have reported that the exosome yield from 3D MSCs cultured in bioreactors is 7.5 times higher than that from 2D MSC cultures [69]. Cao et al. also showed that the exosome yield from 3D MSCs cultured in a hollow-fiber bioreactor was 19.4 times higher than from 2D exosomes [70].

The micro-well array plate method involves seeding cells into small well arrays (typically 100 to 500 µm in diameter), where they spontaneously aggregate to form spheroids. A low-adhesion coating is added to the wells to promote cell self-assembly, and the micro-wells can be square, cylindrical, hexagonal, or other shapes [71]. This method allows the production of more spheroids than the hanging drop method [72]. Recent studies have successfully used spheroids to generate multi-potent stem-like cells under serum-free conditions, and cultured stem cells secreted exosomes that enhanced fibroblast migration and proliferation [73,74]. However, the size of spheroids is often inconsistent, and many cells are lost because they do not participate in spheroid formation.

Magnetic cell levitation has also been developed for spheroid generation [75,76,77,78]. This technique involves loading cells with magnetic nanoparticles and applying an external magnetic field to force cell aggregation to quickly form multicellular spheroids. The resulting spheroids can be easily manipulated because of the magnetic properties of the cells, and the presence of nanoparticles offers advantages for the tracking and imaging of the cells [79]. However, the incorporation of magnetic particles negatively affects cell viability, phenotypic expression, and function [80,81,82]. Further analysis is required to understand the composition of exosomes released from manipulated cells.

Finally, the use of bioprinting to produce spheroids involves ejecting bioink-containing cells into spherical shapes and allowing them to aggregate within the bioink to form spheroids [83]. The automation of bioprinting systems allows for precise control over the amount of bioink dispensed, resulting in highly uniform spheroid size. Modulation of the amount of bioink controls the spheroid size. Automated systems eliminate the need for manual processes, such as the hanging drop method, which leads to the mass production of spheroids. In addition, as the number of bioprinting nozzles installed increases, the number of spheroids produced increases proportionally to the number of nozzles. Jeon et al. successfully produced various cell spheroids using bioink that included gelatin, which liquefies at 37 °C inside hydrogel [83]. Park et al. successfully produced spheroids by using this method [84]. Kim et al. introduced a multi-array spheroid bioprinting technology that made the existing technique scalable [85]. However, the further development of bioprinting technology for practical use is necessary because of its high costs. Exosome secretion from spheroids produced by bioprinting is expected to be higher than from 2D cultures; however, this remains to be explored.

## 3. Isolation and Storage of Exosomes

### 3.1. Isolation

Exosomes are difficult to isolate because they vary in size, content, function, and source, and the purity of exosomes is low because they cannot be separated from lipoproteins and extracellular vesicles derived from the non-endosomal pathway with similar biological properties [4]. Therefore, an efficient method to isolate exosomes is very important for the development of therapeutic technologies utilizing exosomes, and ultracentrifugation, size-based separation technology, polymer precipitation, and immunoaffinity capture technology are mainly used.

Ultracentrifugation is the gold standard for exosome isolation and is currently the most widely used separation technology. This is a technology that uses the difference in sedimentation coefficient according to the size and density of each component mixed in the original solution to separate the required components [86]. First, a series of continuous low-medium speed centrifugation is performed to remove cell debris and large extracellular vesicles; then, exosomes are separated with a centrifugal force of 100,000× *g* and are washed with PBS to remove impurities [87]. However, there are disadvantages such as time consumption, high cost, structural damage, aggregation into blocks, and co-separation of lipoproteins [88,89].

Size-based isolation technology is a method that uses size-exclusion chromatography (SEC) to separate exosomes and other components based on size differences. In SEC, large molecules cannot enter the gel pores and are eluted along the gap between the porous gels, while small molecules remain in the gel pores and are eluted last by the mobile phase [90]. Exosomes separated using SEC have the advantage of having a complete structure and uniform size; however, other particles of similar size may be mixed, which may lower the purity [89].

Polymer precipitation uses polyethylene glycol as a medium to reduce the solubility of exosomes in a solvent and harvest them by centrifugation [90]. The polymer precipitation method has a short analysis time, is relatively easy to operate, and is suitable for processing a large number of samples. However, it has the disadvantage of relatively low purity and recovery rate, and it is difficult to remove the used polymer, making subsequent functional tests difficult [90].

Immunoaffinity chromatography (IAC) is a separation and purification technique based on the specific binding of antibodies and ligands, and the biomarker (antigen) applied by this method must be high-abundance proteins on the surface of the exosome membrane [90]. It has strong specificity, high sensitivity, high purity, and high yield. Compared to ultracentrifugation, IAC can produce similar results with a smaller sample amount [91]. However, it is not suitable for isolating exosomes in large quantities.

### 3.2. Storage

Because exosomes cannot be stored for a long time, it is necessary to study exosome preservation techniques for convenient transportation and clinical application. The preservation techniques currently used mainly include freezing, freeze-drying, and spray-drying.

Cryopreservation is a method to maintain the functional stability of exosomes by lowering the temperature below the temperature required for biochemical reactions to maintain the functional stability of exosomes and is generally applied at temperatures of 4 °C, −80 °C, and −196 °C [90]. Since “frostbite” is likely to occur during the cryopreservation process, an appropriate concentration of antifreeze is selectively added to extend the storage life [92,93,94]. Permeable antifreezes such as dimethyl sulfoxide (DMSO) and ethylene glycol that penetrate into cells can prevent the formation of ice crystals, and it is known that short-term cryopreservation using DMSO for less than 2 months does not significantly change the function of exosomes [95]. Non-permeable antifreezes include trehalose, sucrose, and other carbohydrates. Considering safety, trehalose is known as the most effective disaccharide antifreeze [96].

Freeze-drying is a technique that changes a moisture-contained material to a solid by freezing then directly sublimates and removes the ice in the material under a vacuum to complete the dehydration and drying of the product. The material can be easily stored and can be reconstituted by simply adding water. During the freeze-drying process, the molecular structure of biomolecules can be destroyed due to changes in temperature and pressure; therefore, a type of antifreeze is added to improve the preservation performance [97].

Spray-drying is a technique that sprays an extracellular vesicles solution in a drying room and rapidly evaporates the solvent in contact with hot air to obtain a dried powder. In this technique, the spray pressure and outlet temperature affect the exosome stability, and the product particle size can be controlled [94]. To date, the most widely used storage method is −80 °C freezing; however, there have been reports that the biological activity of exosomes decreases during 28 days of storage at −80 °C [98]. Therefore, studies on the long-term storage stability of exosomes are also thought to be necessary.

## 4. Utilization of Bioprinting in Exosome-Related Regenerative Medicine

### 4.1. Bioprinting

**Figure 2:**
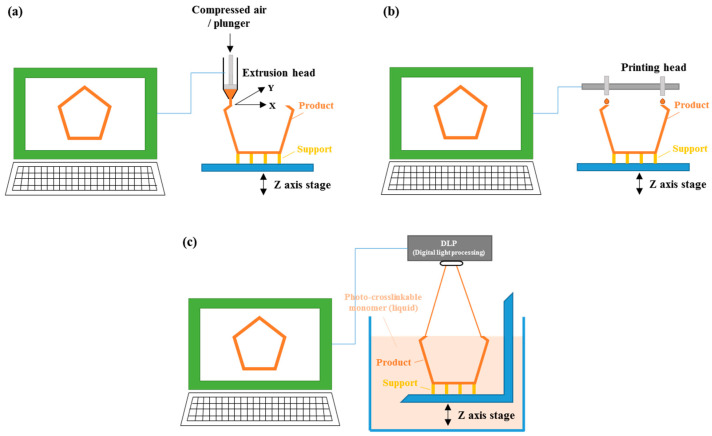
Bioprinting processes for exosome-related regenerative medicine: (**a**) Extrusion-based bioprinting, (**b**) Inkjet-based bioprinting, (**c**) Stereolithography bioprinting (Adapted with permission from Ref. [99]).

Printing technology, which creates 3D structures by sequentially stacking 2D pattern layers, has shown significant potential in the field of regenerative medicine, particularly when combined with medical imaging data. One of its notable applications is bioprinting, which integrates cells and growth factors into a bioink, using a precisely controlled three-axis stage to print 3D structures. This technology has gained attention as a promising method for the regeneration of soft and complex tissue regeneration [99]. With technological advances, significant demonstrations have been made in various fields such as bone engineering, artificial blood vessels, nerve injury treatment, and skin regeneration [100,101,102,103]. However, to improve tissue reconstruction similar to that of the original human tissues or organs, the supply of more cells and biomolecules for organizing the printed cells is essential. The use of spheroids and the application of exosomes secreted from spheroids are drawing attention as technologies that satisfy both cell organization and biomolecule supply. Among the bioprinting techniques studied, the following are primarily used: (1) extrusion-based bioprinting, which controls the deposition by moving the x-y-z axis stage while simultaneously extruding thermoplastic polymers or hydrogels through a nozzle; (2) inkjet-based bioprinting, which creates 3D structures by placing low-viscosity bioink containing cells and exosomes in designated positions; and (3) stereolithography bioprinting, which solidifies specific patterns through light exposure in liquid polymers containing photo-initiators (Figure 2).

### 4.2. Exosome-Loaded Bioprinting

During bioprinting, the cells require assistance from biomolecules or their surrounding environment to organize more quickly into tissues. To create a favorable environment for the target tissue, researchers have experimented with using a tissue-derived extracellular matrix as a printing material or combining various types of cells that constitute the tissue for bioprinting. Additionally, growth factors such as bone morphogenetic protein (BMP), transforming growth factor beta (TGF-β), vascular endothelial growth factor (VEGF), and fibroblast growth factor (FGF), which activate tissue reconstruction, have been utilized. Biomolecules extracted from cultured cells, instead of refined growth factors, have been reported to reduce adverse reactions and are more cell-friendly [104,105,106]. This chapter summarizes the studies that have directly applied exosome-loaded bioinks for tissue reconstruction (Table 1).

#### 4.2.1. Bone Regeneration

Bone grafting is a common approach for treating bone tissue loss; however, there is a shortage of supply relative to demand. To address this issue, tissue-engineered artificial bone construction using techniques such as bioprinting has gained attention. However, owing to the limited biological activity of many existing studies aimed at bone restoration [107], loading exosomes into bioinks for bone tissue regeneration has become a practical option [108]. Yerneni et al. demonstrated that BMP-2 loaded into engineered BMP2-exosomes (eBMP2-ex) using inkjet printing technology can regulate osteoblastogenesis [109]. Additionally, although both eBMP2-ex and free BMP-2 similarly regulate osteoblast formation, eBMP2-ex can maintain this effect over a longer period, inducing osteogenesis both in vitro and in vivo. This was attributed to the ability of eBMP2-ex to bypass cell surface receptors and deliver BMP-2 directly into the cytoplasm. Chen et al. mixed BMSCs secreted exosomes with a decellularized cartilage ECM/gelatin methacrylate bioink and used dynamic projection stereolithography in the visible light spectrum to fabricate 3D scaffolds [110]. After implanting these scaffolds into a rabbit osteochondral defect model, the exosomes released from the scaffolds enhanced cartilage regeneration by rescuing mitochondrial dysfunction in chondrocytes and promoting chondrocyte migration in vivo.

Zha et al. produced porous scaffolds using extrusion-based 3D printing by attaching exosomes loaded with vascular endothelial growth factor-encoding genes to the scaffold for vascularized bone remodeling [104]. Twelve weeks after the implantation of the exosome-activated scaffold into a rat defect model and micro-CT analysis, it was confirmed that the newly formed bone had integrated well with the original tissue. Hematoxylin and eosin staining confirmed the presence of new blood vessels, and immuno-fluorescence staining showed positive results for the angiogenic marker CD31. Bari et al. evaluated the potential for bone tissue regeneration by directly adsorbing the lyosecretome, a lyophilized formulation of the adipose-derived MSC secretome containing exosomes and proteins, onto the surface of 3D printed biodegradable polymer structures and integrating it into alginate bioinks for co-printing with polymers [105]. The addition of mannitol to the control medium before lyophilization preserved the integrity of the exosomes and stabilized the secreted proteins. A polycaprolactone (PCL) scaffold prepared using the adsorption approach showed a rapid release of exosomes and proteins, whereas, in a composite scaffold composed of PCL and alginate hydrogel, the release of exosomes and proteins could be controlled by adjusting the composition and crosslinking density of the alginate hydrogel.

Zhang et al. developed 3D-printed polylactic acid (PLA) scaffolds with improved osteogenic and immunomodulatory potential by incorporating exosomes [111]. After isolating exosomes from human bone marrow stem cells (BMSCs), porous 3D-printed PLA scaffolds were coated with polydopamine to induce exosome adhesion. The exosome-loaded scaffold exhibited high biocompatibility and immunomodulatory potential by reducing the expression of inflammatory markers and the production of reactive oxygen species (ROS). In addition, the scaffold enhanced the osteogenic differentiation. Sun et al. noted that bioceramics play a crucial role in macrophage immunomodulation, and they developed a porous scaffold using β-tricalcium phosphate (β-TCP) bioceramic combined with alginate and hyaluronic acid bioinks after generating exosomes from macrophages using 3D bioprinting technology [112]. The 3D bioprinted exosome scaffold exhibited significant immunomodulatory effects and enhanced osteogenesis and angiogenesis through sustained exosome release. The scaffold also promoted osteogenic differentiation and immunosuppression of BMSCs while enhancing angiogenic activity in human umbilical vein endothelial cells in vitro.

Wu et al. investigated the effects of Schwann cells (SCs) and SC-derived exosomes on bone tissue regeneration by combining SC exosomes with porous Ti6Al4V scaffolds [113]. They added SC-derived exosomes to BMSC cultures and observed that the group containing the exosomes exhibited increased cell proliferation, migration, and osteogenic differentiation in vitro. The cellular activity was further enhanced in the group with higher exosome concentrations (10^8^ particles/mL) than in the group with lower concentrations (10^7^ particles/mL). Additionally, it was confirmed that exosome incorporation enhanced the efficacy of titanium alloy scaffolds in bone repair.

#### 4.2.2. Vessel Regeneration

Vessel formation is essential for obtaining clinically relevant tissue volumes and ultimately fabricating tissues and organs suitable for transplantation. Three-dimensional bioprinting can also apply exosomes to enhance the biological activity necessary for vascular reconstruction. Born et al. demonstrated that MSC-derived exosomes can be integrated into a methacrylate hydrogel bioink for 3D printing [114]. Increasing the concentration of lithium phenyl-2,4,6-trimethylbenzoylphosphinate, a UV crosslinker, during the gelation reduced the initial burst release of exosomes. Additionally, they confirmed through an endothelial gap closure assay that the exosomes retained their biological activity even after the 3D printing and photocrosslinking processes. Since this assay has been shown in their previous research to correlate with in vivo angiogenic promotion, it demonstrated the therapeutic potential of a gelatin methacrylate (GelMA) bioink containing MSC-derived exosomes. Maiullari et al. applied exosomes to angiogenesis by collecting exosomes from human vascular endothelial cells cultured under various stress conditions and using them as additives in bioinks [115]. After analyzing the exosomes obtained under different stress conditions, bioprinted GelMA–alginate structures loaded with exosomes were subcutaneously implanted into mice. These results confirm that the exosome-loaded bioprinted structures supported the formation of new functional blood vessels. They also observed that exosomes derived from human vascular endothelial cells cultured under hypoxic conditions in a serum-free medium induced the highest level of vascular maturation.

#### 4.2.3. Nerve Regeneration

Nerve damage, including injuries to the central and peripheral nervous systems, has a high incidence rate and unclear pathogenesis, with many cases lacking clear treatment strategies [116]. Recently, many researchers have explored the potential of 3D printing and exosomes for nerve repair. Liu et al. developed a 3D printed collagen/silk fibroin scaffold loaded with hypoxia-preconditioned human umbilical cord mesenchymal stem cell (HUCMSC)-derived exosomes (3D-CSHMExos) to regenerate brain defects after traumatic brain injury (TBI) [117]. When 3D-CSHMExos were implanted into the damaged brains of beagle dogs, biocompatibility, neural regeneration, and angiogenesis were improved. Moreover, the 3D-CSHMExos scaffold suppressed neuronal apoptosis and the expression of pro-inflammatory factors such as TNF-α and IL-6, while promoting the expression of the anti-inflammatory factor IL-10, leading to enhanced recovery of motor function after TBI. Liu et al. investigated the role of scaffold stiffness in hydrogels loaded with MSC-derived exosomes during nerve regeneration [118]. In experiments using photocrosslinked hyaluronic acid methacrylate hydrogels, softer hydrogels facilitated the rapid release of exosomes, which suppressed the expression of IL-1β and TNF-α in damaged nerves and promoted peripheral nerve repair. This demonstrates that the stiffness of exosome-loaded hydrogels can control the release rate and pattern of exosomes, thereby offering insights into the use of scaffold materials for clinical applications.

#### 4.2.4. Muscle Regeneration

As most organs in the body require movement to work properly, certain parts of the organs contain muscle tissue. Therefore, an understanding of muscle function and the reconstruction of related tissues is essential for the reconstruction of functional tissues and organs. Exosomes have been studied to aid muscle tissue regeneration. Yerneni et al. introduced macrophage-derived exosomes into inkjet-based bioprinting to create scaffolds and investigated their effects on C2C12 mouse myoblasts [106]. To facilitate bioprinting, glycerol was added to reduce exosome aggregation and increase the bioink viscosity. The exosomes involved in printing were absorbed by C2C12 cells within 15 min. Exosomes derived from inflammatory M1 macrophages spatially inhibited muscle generation, whereas exosomes from regenerative M2 macrophages promoted a microenvironment conducive to muscle generation, thereby inducing myogenesis.

#### 4.2.5. Others

To date, the use of exosome-based bioprinting technologies in cancer treatment has not been systematically studied. However, animal experiments have shown promising potential, and bioprinting can easily supply multiple homogeneous samples, making exosomes highly promising biomarkers for diagnostic purposes. Yerneni et al. proposed a novel method using rapidly scalable oligonucleotide tethers that enables surface functionalization of human and rat exosomes [119]. This exosome surface modification tool can easily and efficiently enhance various natural characteristics of exosomes, ranging from reactive functional groups and small molecules to aptamers and large proteins. Immunomodulatory protein functionalized exosomes, when bioprinted into a collagen matrix, allow spatial for the induction of cell death in tumor cells during printing and inhibit the proliferation of alloreactive T cells when injected into mice. Theodoraki et al. monitored the responses of 18 patients treated for head and neck squamous cell carcinoma (HNSCC) using circulating tumor-derived exosomes (TEX) and T-cell-derived exosomes instead of immune cells [120]. In patients with disease recurrence, the total exosome protein, TEX/total exosome ratio, and total regulatory T cell-derived (CD3+, CD3(-)PD-L1+, and CD3+ CD15s+) exosomes increased from baseline levels. In disease-free patients, total exosome protein and TEX levels decreased, while CD3+ and CD3+ CD15s+ exosomes stabilized, and CD3+ CTLA4+ exosomes decreased after treatment with ipilimumab. This demonstrates the potential role of exosomes as tumor biomarkers.

**Table 1:** Bioprinting studies using exosomes.

Authors (Year)	Utilization	Target	Printing Method	Printing Materials
Yerneni et al. (2021) [109]	Engineered BMP2-exosomes	Bone	Inkjet-based printing	Aellular dermal matrix
Chen et al. (2019) [110]	BMSCs derived exosomes	Bone and cartilage	Dynamic projection stereolithography	Decellularized cartilage ECM/gelatin methacrylate
Zha et al. (2021) [104]	Vascular endothelial growth factor-encoding genes loaded exosomes	Bone and vessel	Extrusion-based bioprinting	Polycaprolactone
Bari et al. (2021) [105]	Adipose derived MSC secretomes	Bone	Extrusion-based bioprinting	Polycaprolactone
Zhang et al. (2021) [111]	BMSCs derived exosomes	Bone	Extrusion-based bioprinting	Polylactic acid
Sun et al. (2022) [112]	Macrophages derived exosomes	Bone and vessel	Extrusion-based bioprinting	β-tricalcium phosphate (β-TCP)/alginate/hyaluronic acid
Wu et al. (2020) [113]	Schwann cells (SCs) derived exosomes	Bone	Electron beam-melting 3D printing	Ti6Al4V
Born et al. (2022) [114]	MSC derived exosomes	Vessel	Stereolithography	Gelatin methacrylate
Maiullari et al. (2021) [115]	Human vascular endothelial cells derived exosomes	Vessel	Extrusion-based bioprinting	Gelatin methacrylate/alginate
Liu X. et al. (2022) [117]	Human umbilical cord mesenchymal stem cell-derived exosomes	Nerve and vessel	Extrusion-based bioprinting	Collagen/silk fibroin
Liu Z. et al. (2022) [118]	Human umbilical cord mesenchymal stem cell-derived exosomes	Nerve	Photocrosslinking	hyaluronic acid methacrylate
Yerneni et al. (2019) [106]	Macrophage-derived exosomes (M1 and M2)	Muscle	Inkjet-based bioprinting	Glycerol
Yerneni et al. (2019) [119]	Immunomodulatory protein functionalized exosomes	Tumor cells	Inkjet-based bioprinting	Glycerol
Theodoraki et al. (2019) [120]	Tumor-derived exosomes (TEX) and T-cell derived exosomes	Cancer diagnosis	Inkjet-based bioprinting	Glycerol

### 4.3. Spheroid-Loaded Bioprinting

Since Mironov’s initial efforts to bioprint spheroids and investigate their potential for vascularization [121,122], numerous examples of spheroid usage in bioprinting have emerged. This chapter summarizes studies focusing on spheroid bioprinting combined with the release of cell-secreted products, such as exosomes (Table 2).

**Figure 3:**
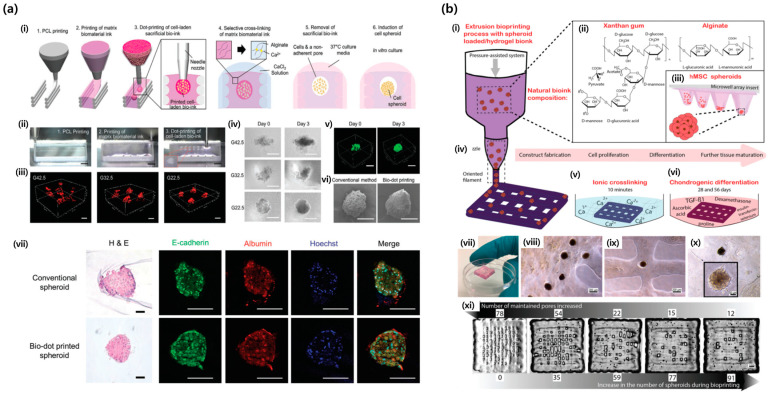
(**a**) Three-dimensional bio-dot printing process with in situ formation of cell spheroids: (i) Schematic illustration showing the bio-dot printing procedure from PCL printing to induction of cell spheroids. (ii) Corresponding still images of the dot-printing process with PCL, matrix biomaterial ink, and cell-laden bio-ink (scale bars, 1 mm). (iii) Three-dimensional confocal images of constructs bio-dot-printed with red fluorescent micro-beads-laden sacrificial bio-ink at varying gelatin concentrations within matrix biomaterial ink: from 22.5 mg mL^−1^ (G22.5) to 42.5 mg mL^−1^ (G42.5). (iv) Microscopy images of HepG2 cells cultured for 3 days after bio-dot printing (scale bars, 200 μm). (v) Three-dimensional confocal images of cells stained with calcein-AM on day 0 and day 3 after printing (scale bars, 200 μm). (vi) SEM images of HepG2 spheroids produced through the conventional method and bio-dot printing process (scale bars, 200 μm). (vii) Histochemical and mmunocytochemical staining of the spheroids on day 5 (scale bars, 100 μm) (“Reprinted with permission from Ref. [83]. Copyright 2020 Wiley-VCH GmbH”). (**b**) Extrusion bioprinting process with the spheroid-loaded bioink. (i) Bioprinting method to form 3D constructs by the deposition of cell spheroids, suspended in a homogeneous printable hydrogel. (ii) The bioink was composed of mixtures of xanthan gum and alginate, capable of crosslinking with Ca^2+^ ions. (iii) HMSC spheroids were obtained by culturing cells in a 3D microenvironment in non-cell-adhesive agarose hydrogel array inserts. (iv) By applying controlled pressure, the bioink was extruded through the nozzle in the form of a filament on pre-designed patterns to manufacture the constructs. (v) Constructs with proliferating cells were ionically crosslinked using calcium chloride and, subsequently, (vi) differentiated using a chondrogenic medium over 28 and 56 days to obtain chondral constructs. (vii) Typical visual aspect of 4-layered bioprinted constructs made of hMSC spheroids and XG3.75:A1.12 hydrogel. (viii) Spheroids maintained their 3D conformational structure 7 days after bioprinting. (ix) Size proportion between printed filaments and hMSC spheroid incorporated in the hydrogel 7 days after bioprinting. (x) Detachment of some cells from the 3D spheroid structure toward the hydrogel. (xi) In a clear trend, the open-pore structure was gradually reduced as the number of printed spheroids per construct increased (“Reprinted with permission from Ref. [126]. Copyright 2023 Wiley-VCH GmbH”).

Jeon et al. developed a new spheroid bioprinting technology called 3D bio-dot printing, which allows the precise creation of 3D patterns with various cell spheroids [83] (Figure 3a). This technology created non-adhesive micro-pores within 3D structures to induce the formation of cell spheroids. Their work realized in situ formation of different cell spheroids, including hepatocytes, pancreatic β-cells, and breast cancer cells, and allows for the printing of 3D structures with multiple spheroid types. The usefulness of this technology for improving liver function and drug metabolism has been demonstrated. Han et al. applied this technique to create in vitro breast cancer models that accurately mimicked patient-specific cancer morphologies [123]. This method generates cancer cell spheroids with ductal and solid microstructures, reflecting different stages of breast cancer. The bioprinted models exhibited genotypic and phenotypic characteristics, including hypoxia, invasion, and drug resistance, similar to those of human cancers. Kim et al. precisely bioprinted decellularized extracellular matrix (dECM)-incorporated hepatocyte spheroids with diameters of approximately 160–220 μm using primary mouse hepatocytes (PMH) [124]. Compared with hepatocyte-only spheroids, dECM-incorporated hepatocyte spheroids exhibited approximately 4.3-fold and 2.5-fold increased albumin and urea secretion levels, respectively, a 2.0-fold increase in CYP enzyme activity, and up to 1.8-fold enhanced drug reactivity to hepatotoxic drugs. Their spheroid printing technique showed great potential for the development of a highly functional in vitro liver tissue model for drug toxicity assessment. Similarly, Park et al. developed a precise and efficient method for printing spheroidal multicellular microarchitectures (SMMs) that created a tissue-specific microenvironment, potentially useful for cell therapy [85]. This method uses a bioink blended with dECM and alginate to enhance the cell function. The experimental results demonstrated the controllable size, mass production, and high cell viability of the SMMs. Co-culturing SMMs with endothelial cells improves maturation and functionality, showing promise for tissue regeneration, particularly in the treatment of myocardial infarction, compared with single-cell injections. They also proposed a single-step bioprinting method using a dual-crosslinkable decellularized extracellular matrix with ruthenium/sodium persulfate (dERS) [125]. Using this method, spheroids and tubular structures were fabricated without multiple post-processing steps.

Decarli et al. proposed a reproducible bioprinting process using human mesenchymal stromal cell spheroids in a xanthan gum alginate hydrogel to create stable multi-layered constructs [126] (Figure 3b). After 28 d of chondrogenic differentiation and 56 d of culture, the constructs showed good stability, cell activity, and extracellular matrix production, indicating their potential as cartilage implants.

Kim et al. introduced a high-throughput integrated tissue formation system for bioprinting (HITS-Bio), that enabled scalable tissue fabrication by rapidly deploying multiple spheroids simultaneously using a digitally controlled nozzle array (DCNA) [127]. This system demonstrated the ability to bioprint microRNA-transfected spheroids (approximately 30 mm^3^) to regenerate cranial bone in a rat model, achieving near-complete defect closure (approximately 91% after 3 weeks and 96% after 6 weeks). Additionally, HITS-Bio was used to construct a scalable cartilage structure (1 cm^3^) consisting of approximately 600 cartilage spheroids to repair volumetric tissue defects.

## 5. Prospects for the Development of Bioprinting Technology Utilizing Exosomes

Exosomes, first discovered in reticulocytes in 1983 and initially thought to be cellular waste products [128,129,130], have garnered significant public interest as carriers of genetic information since it was found that cells could exchange genetic material via RNA through exosomes [131]. Since then, researchers have identified a wide variety of exosomes with diverse functions and have highlighted their immense potential for tissue regeneration, disease diagnosis, and treatment. Bioprinting, derived from 3D printing technology, enables the application of cells, proteins, DNA, and other biological materials into personalized 3D models or functional biological structures. This computer-aided design (CAD)-driven bioprinting has shown great potential for replicating the complexity of native tissues in terms of mechanical properties, specific structures, and interactions between cells and the extracellular matrix (ECM) [132,133,134]. The convergence of these two promising technologies, bioprinting, and exosomes, has great potential for advancing medical and regenerative medicine. The prospects for bioprinting technology utilizing exosomes are as follows:

(1) Precision cell therapy and tissue regeneration: Exosomes are secreted by various cell types and have distinct regenerative capabilities. Bioprinting technology, which enables precise control of positioning in 3D space, allows for the accurate distribution and layering of exosomes to maximize cell therapy and tissue regeneration [135,136]. As most organs in the human body consist of multi-layered complex tissues, delivering tailored exosomes suited to the reconstruction of damaged tissues can suppress inflammation and abnormal reactions; thus, promoting tailored regenerative treatments.

(2) Tissue-specific exosome production: Current exosome research focuses on the production of exosomes from specific cells or stem cells [57]. The use of spheroids, a type of 3D cell culture, in bioprinting offers a powerful tool for arranging various cell types in forms similar to those in the human body, allowing for the cultivation of exosomes tailored to specific target tissues. In particular, in clinical applications requiring the reconstruction of volumetric tissues, the proper formation and networking of blood vessels composed of endothelial cells, muscles, and connective tissue can significantly expand the possibilities of personalized tissue engineering and organ transplantation [115].

(3) Exosome-loaded scaffold development: By utilizing bioprinting technology with spheroid aggregates, 3D structures that mimic the extracellular matrix (ECM) can be created to optimize exosome delivery [137]. Incorporating nanostructures that can capture exosomes or adjust hydrogel structures to allow for the continuous release of exosomes at specific sites could enable sustained and effective exosome delivery that surpasses conventional intravenous or localized injection methods [138,139,140,141]. This scaffold technology is expected to play a crucial role, particularly in tissue regeneration areas where form and structural integrity are vital, such as bone and cartilage regeneration.

(4) Personalized medicine and customized therapy: Combining exosomes derived from patient cells with bioprinting technology can pave the way for personalized treatment. To date, exosomes have been used in cancer treatment to analyze outcomes and track disease recurrence, with experiments on rodent models demonstrating the induction of tumor cell death [119,120]. However, with continued research, it may be possible to extract functionalized exosomes from bioprinted spheroids to perform targeted therapy that kills only cancer cells, or to perform immunotherapy by obtaining patient-specific T cell-based exosomes. Even if it does not have those treatment levels, it will be possible to suggest improved cancer treatment methods using patient-specific anticancer drug selection and multiple anticancer drug combinations by analyzing exosomes secreted from bioprinted patient-derived 3D cancer spheroids in the near future. This represents a shift from the current standardized treatment to a precision medicine strategy tailored for individual patients.

(5) Industrial Expansion: The development of exosomes and bioprinting technologies also presents significant potential for industrial expansion in the medical field. Currently, the most efficient and easy-to-use method for producing spheroids is to use 96-well microplates with ultra-low attachment surfaces [142]. However, this has the disadvantages of high cost and unreliability due to manual labor for mass production. Bioprinting-based automation systems that eliminate manual work lead to the mass production of spheroids. As the number of bioprinting nozzles installed increases, the number of spheroids produced increases proportionally to the number of nozzles. If automated bioprinters could produce large quantities of high-purity exosomes, it could lead to the commercialization of exosome-based therapeutics and bioprinted products for regenerative medicine [143]. Exosome-based treatments produced through 3D bioprinting can revolutionize the medical, pharmaceutical, and medical device industries, transforming treatment paradigms for various diseases. Moreover, the use of exosomes in the cosmetic industry will open up a new market [144,145,146].

However, several challenges remain to be addressed, such as spheroid culture, exosome isolation, purification, long-term storage and packaging, the determination of appropriate usage concentrations, and the establishment of optimal dosing periods and frequencies [147].

## 6. Conclusions

This review discussed recent advances in the utilization of exosomes and spheroids in bioprinting as a promising approach in regenerative medicine. First, we explored the definition of exosomes and their potential applications in tissue regeneration, disease diagnosis, and therapeutics. In addition, we highlighted spheroids among the various cell culture methods for exosome production, which are suitable for large-scale production and can be used in bioprinting. Furthermore, we provided an overview of research findings on the application of spheroids and exosomes in bioprinting and discussed the prospects of bioprinting technology using exosomes. While there are currently limited results on the direct use of bioprinted spheroids as exosome-secreting sources for tissue engineering, we speculate that if a large number of spheroids can be supplied through bioprinting, the spheroid-exosome-based bioprinting technology will provide new possibilities for application in tissue regeneration, disease diagnosis, and treatment.

## Figures and Tables

**Table 2:** Bioprinting studies using spheroids.

Authors (Year)	Utilization	Target	Printing Method	Printing Materials
Jeon et al. (2020) [83]	Spheroids, including hepatocytes, pancreatic β-cells, and breast cancer cells	Various tissue	Extrusion-based bioprinting	Collagen/gelatin/alginate
Han et al. (2022) [123]	Bioprinted spheroids for exosome secretion	Breast cancer	Extrusion-based bioprinting	Collagen/alginate/Hyaluronic acid
Kim et al. (2023) [124]	Hepatocyte spheroids	Liver	Extrusion-based bioprinting	Decellularized extracellular matrix/hyaluronic acid/gelatin
Park et al. (2021) [84]	Spheroidal multicellular microarchitectures with endothelial cells	Muscle	Extrusion-based bioprinting	Decellularized extracellular matrix/alginate
Kim et al. (2022) [125]	Decellularized extracellular matrix	Vessel	Extrusion-based bioprinting	Decellularized extracellular matrix
Decarli et al. (2023) [126]	Mesenchymal stromal cell spheroids	Cartilage	Extrusion-based bioprinting	Xanthan gum-alginate
Kim et al. (2024) [127]	MicroRNA-transfected spheroids	Bone and cartilage	Extrusion-based bioprinting	Gelatin methacryloyl/fibrinogen/hyaluronic acid

## Data Availability

The original contributions presented in the study are included in the article, further inquiries can be directed to the corresponding author.

## References

[1] Ghasroldasht M.M., Seok J., Park H.S., Ali F.B.L., Al-Hendy A. (2022). Stem cell therapy: From idea to clinical practice. Int. J. Mol. Sci..

[2] Hoang D.M., Pham P.T., Bach T.Q., Ngo A.T.L., Nguyen Q.T., Phan T.T.K., Nguyen G.H., Le P.T.T., Hoang V.T., Forsyth N.R. (2022). Stem cell-based therapy for human diseases. Signal Transduct. Target Ther..

[3] Lee A.S., Tang C., Rao M.S., Weissman I.L., Wu J.C. (2013). Tumorigenicity as a clinical hurdle for pluripotent stem cell therapies. Nat. Med..

[4] Kalluri R., LeBleu V.S. (2020). The biology, function, and biomedical applications of exosomes. Science.

[5] Gill S., Catchpole R., Forterre P. (2018). Extracellular membrane vesicles in the three domains of life and beyond. FEMS Microbiol. Rev..

[6] Whiteside T.L. (2016). Tumor-Derived Exosomes and Their Role in Cancer Progression. Adv. Clin. Chem..

[7] Feng Z.-Y., Zhang Q.-Y., Tan J., Xie H.-Q. (2021). Techniques for increasing the yield of stem cell-derived exosomes: What factors may be involved?. Sci. China Life Sci..

[8] Rashed M.H., Bayraktar E., Helal G.K., Abd-Ellah M.F., Amero P., Chavez-Reyes A., Rodriguez-Aguayo C. (2017). Exosomes: From Garbage Bins to Promising Therapeutic Targets. Int. J. Mol. Sci..

[9] Selvam S., Thomas M.B., Bhowmick T., Chandru A. (2023). Bioprinting of exosomes: Prospects and challenges for clinical applications. Int. J. Bioprint..

[10] Zhang Q., Jeppesen D.K., Higginbotham J.N., Graves-Deal R., Trinh V.Q., Ramirez M.A., Sohn Y., Neininger A.C., Taneja N., McKinley E.T. (2021). Supermeres are functional extracellular nanoparticles replete with disease biomarkers and therapeutic targets. Nature.

[11] Fu S., Zhang Y., Li Y., Luo L., Zhao Y., Yao Y. (2020). Extracellular vesicles in cardiovascular diseases. Cell Death Discov..

[12] Kalra H., Drummen G.P., Mathivanan S. (2016). Focus on extracellular vesicles: Introducing the next small big thing. Int. J. Mol. Sci..

[13] Gurung S., Perocheau D., Touramanidou L., Baruteau J. (2021). The exosome journey: From biogenesis to uptake and intracellular signalling. Cell Commun. Signal..

[14] Phuyal S., Hessvik N.P., Skotland T., Sandvig K., Llorente A. (2014). Regulation of exosome release by glycosphingolipids and flotillins. FEBS J..

[15] Li P., Kaslan M., Lee S.H., Yao J., Gao Z. (2017). Progress in Exosome Isolation Techniques. Theranostics.

[16] Li I., Nabet B.Y. (2019). Exosomes in the tumor microenvironment as mediators of cancer therapy resistance. Mol. Cancer.

[17] Huang X., Yuan T., Tschannen M., Sun Z., Jacob H., Du M., Liang M., Dittmar M.R., Liu Y., Liang M. (2013). Characterization of human plasma-derived exosomal RNAs by deep sequencing. BMC Genom..

[18] Hade M.D., Suire C.N., Suo Z. (2021). Mesenchymal stem cell-derived exosomes: Applications in regenerative medicine. Cells.

[19] Herrmann I.K., Wood M.J.A., Fuhrmann G. (2021). Extracellular vesicles as a next-generation drug delivery platform. Nat. Nanotechnol..

[20] Ciferri M., Quarto R., Tasso R. (2021). Extracellular vesicles as biomarkers and therapeutic tools: From pre-clinical to clinical applications. Biology.

[21] Luo R., Liu M., Tan T., Yang Q., Wang Y., Men L., Zhao L., Zhang H., Wang S., Xie T. (2021). Emerging significance and therapeutic potential of extracellular vesicles. Int. J. Biol. Sci..

[22] Limongi T., Susa F., Dumontel B., Racca L., Perrone Donnorso M., Debellis D., Cauda V. (2021). Extracellular vesicles tropism: A comparative study between passive innate tropism and the active engineered targeting capability of lymphocyte-derived evs. Membranes.

[23] Muthu S., Bapat A., Jain R., Jeyaraman N., Jeyaraman M. (2021). Exosomal therapy—A new frontier in regenerative medicine. Stem Cell Investig..

[24] Fuloria S., Subramaniyan V., Dahiya R., Dahiya S., Sudhakar K., Kumari U., Sathasivam K., Meenakshi D., Wu Y., Sekar M. (2021). Mesenchymal stem cell-derived extracellular vesicles: Regenerative potential and challenges. Biology.

[25] Yin K., Wang S., Zhao R.C. (2019). Exosomes from mesenchymal stem/stromal cells: A new therapeutic paradigm. Biomark. Res..

[26] Ma Y., Ge S., Zhang J., Zhou D., Li L., Wang X., Su J. (2017). Mesenchymal stem cell-derived extracellular vesicles promote nerve regeneration after sciatic nerve crush injury in rats. Int. J. Clin. Exp. Pathol..

[27] Lai J.J., Chau Z.L., Chen S., Hill J.J., Korpany K.V., Liang N., Lin L., Lin Y., Liu J.K., Liu Y. (2022). Exosome processing and characterization approaches for research and technology development. Adv. Sci..

[28] Sumrin A., Moazzam S., Khan A.A., Ramzan I., Batool Z., Kaleem S., Ali M., Bashir H., Bilal M. (2018). Exosomes as biomarker of cancer. Braz. Arch. Biol. Technol..

[29] Tai Y.-L., Chen K.-C., Hsieh J.-T., Shen T.-L. (2018). Exosomes in cancer development and clinical applications. Cancer Sci..

[30] Osaki M., Okada F. (2019). Exosomes and their role in cancer progression. Yonago Acta Medica.

[31] Li X., Corbett A.L., Taatizadeh E., Tasnim N., Little J.P., Garnis C., Daugaard M., Guns E., Hoorfar M., Li I.T.S. (2019). Challenges and opportunities in exosome research—Perspectives from biology, engineering, and cancer therapy. APL Bioeng..

[32] D’Anca M., Fenoglio C., Serpente M., Arosio B., Cesari M., Scarpini E.A., Galimberti D. (2019). Exosome determinants of physiological aging and age-related neurodegenerative diseases. Front. Aging Neurosci..

[33] Dardet J.P., Serrano N., András I.E., Toborek M. (2022). Overcoming blood-brain barrier resistance: Implications for extracellular vesicle-mediated drug brain delivery. Front. Drug Deliv..

[34] Ebrahimkhani S., Vafaee F., Young P.E., Hur S.S.J., Hawke S., Devenney E., Beadnall H., Barnett M.H., Suter C.M., Buckland M.E. (2017). Exosomal microRNA signatures in multiple sclerosis reflect disease status. Sci. Rep..

[35] Garcia-Contreras M., Shah S.H., Tamayo A., Robbins P.D., Golberg R.B., Mendez A.J., Ricordi C. (2017). Plasma-derived exosome characterization reveals a distinct microRNA signature in long duration Type 1 diabetes. Sci. Rep..

[36] Sun Y., Tao Q., Wu X., Zhang L., Liu Q., Wang L. (2021). The utility of exosomes in diagnosis and therapy of diabetes mellitus and associated complications. Front. Endocrinol..

[37] Liang Y., Duan L., Lu J., Xia J. (2021). Engineering exosomes for targeted drug delivery. Theranostics.

[38] Soliman H.M., Ghonaim G.A., Gharib S.M., Chopra H., Farag A.K., Hassanin M.H., Nagah A., Emad-Eldin M., Hashem N.E., Yahya G. (2021). Exosomes in alzheimer’s disease: From being pathological players to potential diagnostics and therapeutics. Int. J. Mol. Sci..

[39] Martins-Marques T., Pinho M.J., Zuzarte M., Oliveira C., Pereira P., Sluijter J.P.G., Gomes C., Girao H. (2016). Presence of Cx43 in extracellular vesicles reduces the cardiotoxicity of the anti-tumour therapeutic approach with doxorubicin. J. Extracell. Vesicles.

[40] Kim M.S., Haney M.J., Zhao Y., Yuan D., Deygen I., Klyachko N.L., Kabanov A.V., Batrakova E.V. (2018). Engineering macrophage derived exosomes for targeted paclitaxel delivery to pulmonary metastases: In vitro and in vivo evaluations. Nanomedicine.

[41] Li Y.-J., Wu J.-Y., Wang J.-M., Hu X.-B., Cai J.-X., Xiang D.-X. (2019). Gemcitabine loaded autologous exosomes for effective and safe chemotherapy of pancreatic cancer. Acta Biomater..

[42] Qu M., Lin Q., Huang L., Fu Y., Wang L., He S., Fu Y., Yang S., Zhang Z., Zhang L. (2018). Dopamine-loaded blood exosomes targeted to brain for better treatment of Parkinson’s disease. J. Control. Release.

[43] DeJong O.G., Kooijmans S.A.A., Murphy D.E., Jiang L., Evers M.J.W., Sluijter J.P.G., Vader P., Schiffelers R.M. (2019). Drug delivery with extracellular vesicles: From imagination to innovation. Acc. Chem. Res..

[44] Zhang X., Borg E.G.F., Liaci A.M., Vos H.R., Stoorvogel W. (2020). A novel three step protocol to isolate extracellular vesicles from plasma or cell culture medium with both high yield and purity. J. Extracell. Vesicles.

[45] Syromiatnikova V., Prokopeva A., Gomzikova M. (2022). Methods of the large-scale production of extracellular vesicles. Int. J. Mol. Sci..

[46] do Amaral J.B., Rezende-Teixeira P., Freitas V.M., Machado-Santelli G.M. (2011). MCF-7 cells as a three-dimensional model for the study of human breast cancer. Tissue Eng. Part C Methods.

[47] Białkowska K., Komorowski P., Bryszewska M., Miłowska K. (2020). Spheroids as a type of three-dimensional cell cultures—Examples of methods of preparation and the most important application. Int. J. Mol. Sci..

[48] Hahm J., Kim J., Park J. (2021). Strategies to enhance extracellular vesicle production. Tissue Eng. Regen. Med..

[49] Joo H.S., Suh J.H., Lee H.J., Bang E.S., Lee J.M. (2020). Current knowledge and future perspectives on mesenchymal stem cell-derived exosomes as a new therapeutic agent. Int. J. Mol. Sci..

[50] Xie S., Zhang Q., Jiang L. (2022). Current knowledge on exosome biogenesis, cargo-sorting mechanism and therapeutic implications. Membranes.

[51] Knight E., Przyborski S. (2015). Advances in 3D cell culture technologies enabling tissue-like structures to be created in vitro. J. Anat..

[52] Tibbitt M.W., Anseth K.S. (2009). Hydrogels as extracellular matrix mimics for 3D cell culture. Biotechnol. Bioeng..

[53] Heywood H.K., Sembi P.K., Lee D.A., Bader D.L. (2004). Cellular utilization determines viability and matrix distribution profiles in chondrocyte-seeded alginate constructs. Tissue Eng..

[54] Jongpaiboonkit L., King W.J., Lyons G.E., Paguirigan A.L., Warrick J.W., Beebe D.J., Murphy W.L. (2008). An adaptable hydrogel array format for 3-dimensional cell culture and analysis. Biomaterials.

[55] Topman G., Shoham N., Sharabani-Yosef O., Lin F.H., Gefen A. (2013). A new technique for studying directional cell migration in a hydrogel-based three-dimensional matrix for tissue engineering model systems. Micron.

[56] Yu W., Li S., Guan X., Zhang N., Xie X., Zhang K., Bai Y. (2022). Higher yield and enhanced therapeutic effects of exosomes derived from MSCs in hydrogel-assisted 3D culture system for bone regeneration. Biomater. Adv..

[57] Lee D.H., Yun D.W., Kim Y.H., Im G.B., Hyun J., Park H.S., Bhang S.H., Choi S.H. (2023). Various three-dimensional culture methods and cell types for exosome production. Tissue Eng. Regen. Med..

[58] Huang S.W., Tzeng S.C., Chen J.K., Sun J.S., Lin F.H. (2020). A dynamic hanging-drop system for mesenchymal stem cell culture. Int. J. Mol. Sci..

[59] Kim M., Yun H.W., Park D.Y., Choi B.H., Min B.H. (2018). Three-dimensional spheroid culture increases exosome secretion from mesenchymal stem cells. Tissue Eng. Regen. Med..

[60] Giusti I., Poppa G., D’Ascenzo S., Esposito L., Vitale A.R., Calvisi G., Dolo V. (2022). Cancer three-dimensional spheroids mimic in vivo tumor features, displaying “inner” extracellular vesicles and vasculogenic mimicry. Int. J. Mol. Sci..

[61] Mattot V., Raes M.B., Henriet P., Eeckhout Y., Stehelin D., Vandenbunder B., Desbiens X. (1995). Expression of interstitial collagenase is restricted to skeletal tissue during mouse embryogenesis. J. Cell Sci..

[62] De Groot T., Veserat K., Berthier E., Beebe D., Theberge A. (2016). Surface-tension driven open microfluidic platform for hanging droplet culture. Lab Chip.

[63] Chaicharoenaudomrung N., Kunhorm P., Noisa P. (2019). Three-dimensional cell culture systems as an in vitro platform for cancer and stem cell modeling. World J. Stem Cells.

[64] Haraszti R.A., Miller R., Stoppato M., Sere Y.Y., Coles A., Didiot M.C., Wollacott R., Sapp E., Dubuke M.L., Li X. (2018). Exosomes produced from 3D cultures of MSCs by tangential flow filtration show higher yield and improved activity. Mol. Ther..

[65] Foglietta F., Canaparo R., Muccioli G., Terreno E., Serpe L. (2020). Methodological aspects and pharmacological applications of three-dimensional cancer cell cultures and organoids. Life Sci..

[66] Santos J.M., Camões S.P., Filipe E., Cipriano M., Barcia R.N., Filipe M., Teixeira M., Simões S., Gaspar M., Mosqueira D. (2015). Three-dimensional spheroid cell culture of umbilical cord tissue-derived mesenchymal stromal cells leads to enhanced paracrine induction of wound healing. Stem Cell Res. Ther..

[67] Phelan M.A., Gianforcaro A.L., Gerstenhaber J.A., Lelkes P.I. (2019). An air bubble- isolating rotating wall vessel bioreactor for improved spheroid/organoid formation. Tissue Eng. C Methods.

[68] Gloeckner H., Jonuleit T., Lemke H.-D. (2001). Monitoring of cell viability and cell growth in a hollow-fiber bioreactor by use of the dye Alamar Blue. J. Immunol. Methods.

[69] Yan L., Wu X. (2020). Exosomes produced from 3D cultures of umbilical cord mesenchymal stem cells in a hollow-fiber bioreactor show improved osteochondral regeneration activity. Cell Biol. Toxicol..

[70] Cao J., Wang B., Tang T., Lv L., Ding Z., Li Z., Hu R., Wei Q., Shen A., Fu Y. (2020). Three-dimensional culture of MSCs produces exosomes with improved yield and enhanced therapeutic efficacy for cisplatin-induced acute kidney injury. Stem Cell Res. Ther..

[71] Manzoor A.A., Romita L., Hwang D.K. (2021). A review on microwell and microfluidic geometric array fabrication techniques and its potential applications in cellular studies. Can. J. Chem. Eng..

[72] Gidrol X., Fouqué B., Ghenim L., Haguet V., Picollet-D’hahan N., Schaack B. (2009). 2D and 3D cell microarrays in pharmacology. Curr. Opin. Pharmacol..

[73] Faruqu F.N., Zhou S., Sami N., Gheidari F., Lu H., Al-Jamal K.T. (2020). Three-dimensional culture of dental pulp pluripotent-like stem cells (DPPSCs) enhances Nanog expression and provides a serum-free condition for exosome isolation. FASEB BioAdv..

[74] Faruqu F.N., Liam-Or R., Zhou S., Nip R., Al-Jamal K.T. (2021). Defined serum-free three-dimensional culture of umbilical cord-derived mesenchymal stem cells yields exosomes that promote fibroblast proliferation and migration in vitro. FASEB J..

[75] Ho V.H., Guo W.M., Huang C.L., Ho S.F., Chaw S.Y., Tan E.Y., Ng K.W., Loo J.S. (2013). Manipulating magnetic 3D spheroids in hanging drops for applications in tissue engineering and drug screening. Adv. Healthc. Mater..

[76] Olsen T.R., Mattix B., Casco M., Herbst A., Williams C., Tarasidis A., Simionescu D., Visconti R.P., Alexis F. (2015). Manipulation of cellular spheroid composition and the effects on vascular tissue fusion. Acta Biomater..

[77] Mattix B., Olsen T.R., Gu Y., Casco M., Herbst A., Simionescu D.T., Visconti R.P., Kornev K.G., Alexis F. (2014). Biological magnetic cellular spheroids as building blocks for tissue engineering. Acta Biomater..

[78] Mattix B.M., Olsen T.R., Casco M., Reese L., Poole J.T., Zhang J., Visconti R.P., Simionescu A., Simionescu D.T., Alexis F. (2014). Janus magnetic cellular spheroids for vascular tissue engineering. Biomaterials.

[79] Lewis N.S., Lewis E.E., Mullin M., Wheadon H., Dalby M.J., Berry C.C. (2017). Magnetically levitated mesenchymal stem cell spheroids cultured with a collagen gel maintain phenotype and quiescence. J. Tissue Eng..

[80] Ho V.H.B., Müller K.H., Barcza A., Chen R., Slater N.K.H. (2010). Generation and manipulation of magnetic multicellular spheroids. Biomaterials.

[81] Bratt-Leal A.M., Kepple K.L., Carpenedo R.L., Cooke M.T., McDevitt T.C. (2011). Magnetic manipulation and spatial patterning of multi-cellular stem cell aggregates. Integr. Biol..

[82] Akiyama H., Ito A., Kawabe Y., Kamihira M. (2009). Cell-patterning using poly (ethylene glycol)-modified magnetite nanoparticles. J. Biomed. Mater. Res. Part A.

[83] Jeon S., Heo J.H., Kim M.K., Jeong W., Kang H.W. (2020). High-precision 3D bio-dot printing to Improve paracrine interaction between multiple types of cell spheroids. Adv. Funct. Mater..

[84] Park Y., Ji S.T., Yong U., Das S., Jang W.B., Ahn G., Kwon S.M., Jang J. (2021). 3D bioprinted tissue-specific spheroidal multicellular microarchitectures for advanced cell therapy. Biofabrication.

[85] Dai S., Wei D., Wu Z., Zhou X., Wei X., Huang H., Li G. (2008). Phase I clinical trial of autologous ascites-derived exosomes combined with GM-CSF for colorectal cancer. Mol. Ther..

[86] Livshits M.A., Khomyakova E., Evtushenko E.G., Lazarev V.N., Kulemin N.A., Semina S.E., Generozov E.V., Govorun V.M. (2015). Isolation of exosomes by differential centrifugation: Theoretical analysis of a commonly used protocol. Sci. Rep..

[87] Théry C., Amigorena S., Raposo G., Clayton A. (2006). Isolation and characterization of exosomes from cell culture supernatants and biological fluids. Curr. Protoc. Stem Cell Biol..

[88] Yang X.X., Sun C., Wang L., Guo X.L. (2019). New insight into isolation, identification techniques and medical applications of exosomes. J. Control. Release.

[89] Boing A.N., van der Pol E., Grootemaat A.E., Coumans F.A., Sturk A., Nieuwland R. (2014). Single-step isolation of extracellular vesicles by size-exclusion chromatography. J. Extracell. Vesicles.

[90] Zhang Y., Bi J., Huang J., Tang Y., Du S., Li P. (2020). Exosome: A review of its classification, isolation techniques, storage, diagnostic and targeted therapy applications. Int. J. Nanomed..

[91] Zarovni N., Corrado A., Guazzi P., Zocco D., Lari E., Radano G., Muhhina J., Fondelli C., Gavrilova J., Chiesi A. (2015). Integrated isolation and quantitative analysis of exosome shuttled proteins and nucleic acids using immunocapture approaches. Methods.

[92] Bahr M.M., Amer M.S., Abo-El-Sooud K., Abdallah A.N., El-Tookhy O.S. (2020). Preservation techniques of stem cells extracellular vesicles: A gate for manufacturing of clinical grade therapeutic extracellular vesicles and long-term clinical trials. Int. J. Vet. Sci. Med..

[93] Jeyaram A., Jay S.M. (2017). Preservation and storage stability of extracellular vesicles for therapeutic applications. AAPS J..

[94] Kusuma G.D., Barabadi M., Tan J.L., Morton D.A.V., Frith J.E., To L.R. (2018). Protect and to preserve: Novel preservation strategies for extracellular vesicles. Front. Pharmacol..

[95] Wu Y., Deng W., Klinke D. (2015). Exosomes: Improved methods to characterize their morphology, RNA content, and surface protein biomarkers. Analyst.

[96] Bosch S., de Beaurepaire L., Allard M., Mosser M., Heichette C., Chrétien D., Jegou D., Bach J.M. (2016). Trehalose prevents aggregation of exosomes and cryodamage. Sci. Rep..

[97] Charoenviriyakul C., Takahashi Y., Nishikawa M., Takakura Y. (2018). Preservation of exosomes at room temperature using lyophilization. Int. J. Pharm..

[98] Lorincz A.M., Timar C.I., Marosvari K.A., Veres D.S., Otrokocsi L., Kittel Á., Ligeti E. (2014). Effect of storage on physical and functional properties of extracellular vesicles derived from neutrophilic granulocytes. J. Extracell. Vesicles.

[99] Lee H.-W., Lee J.W. (2023). Current status and future outlook of additive manufacturing technologies for the reconstruction of the trachea. J. Funct. Biomater..

[100] Genova T., Roato I., Carossa M., Motta C., Cavagnetto D., Mussano F. (2020). Advances on Bone Substitutes through 3D Bioprinting. Int. J. Mol. Sci..

[101] Norotte C., Marga F.S., Niklason L.E., Forgacs G. (2009). Scaffold-free vascular tissue engineering using bioprinting. Biomaterials.

[102] Gao S., Tang Y., Sun W., Liu Z., Zhao T., Li X., Wang T., Liao G., Xu T., Zheng G. (2023). 3D-bioprinted GelMA nerve guidance conduits promoted peripheral nerve regeneration by inducing trans-differentiation of MSCs into SCLCs via PIEZO1/YAP axis. Mater. Today Adv..

[103] Kim B.S., Lee J.-S., Cho D.-W. (2017). Direct 3D cell-printing of human skin with functional transwell system. Biofabrication.

[104] Zha Y., Li Y., Lin T., Chen J., Zhang S., Wang J. (2021). Progenitor cell-derived exosomes endowed with VEGF plasmids enhance osteogenic induction and vascular remodeling in large segmental bone defects. Theranostics.

[105] Bari E., Scocozza F., Perteghella S., Sorlini M., Auricchio F., Torre M.L., Conti M. (2021). 3D bioprinted scaffolds containing mesenchymal stem/stromal lyosecretome: Next generation controlled release device for bone regenerative medicine. Pharmaceutics.

[106] Yerneni S.S., Whiteside T.L., Weiss L.E., Campbell P.G. (2019). Bioprinting exosome-like extracellular vesicle microenvironments. Bioprinting.

[107] Liang Q., Ma Y., Yao X., Wei W. (2022). Advanced 3D-printing bioinks for articular cartilage. Int. J. Bioprinting.

[108] Fan J., Lee C.S., Kim S., Chen C., Aghaloo T., Lee M. (2020). Generation of small RNA modulated exosome mimetics for bone regeneration. ACS Nano.

[109] Yerneni S.S., Adamik J., Weiss L.E., Campbell P.G. (2021). Cell trafficking and regulation of osteoblastogenesis by extracellular vesicle associated bone morphogenetic protein 2. J. Extracell. Vesicles.

[110] Chen P., Zheng L., Wang Y., Tao M., Xie Z., Xia C., Gu C., Chen C., Qiu P., Mei P. (2019). Desktop-stereolithography 3D printing of a radially oriented extracellular matrix/mesenchymal stem cell exosome bioink for osteochondral defect regeneration. Theranostics.

[111] Sun Y.H., Zhang B.J., Zhai D., Wu C. (2021). Three-dimensional printing of bioceramic-induced macrophage exosomes: Immunomodulation and osteogenesis/angiogenesis. NPG Asia Mater..

[112] Zhang Y., Huo M., Wang Y., Xiao L., Wu J., Ma Y., Zhang D., Lang X., Wang X. (2022). A tailored bioactive 3D porous poly(lactic-acid)-exosome scaffold with osteo-immunomodulatory and osteogenic differentiation properties. J. Biol. Eng..

[113] Wu Z., Pu P., Su Z., Zhang X., Nie L., Chang Y. (2020). Schwann cell-derived exosomes promote bone regeneration and repair by enhancing the biological activity of porous Ti6Al4V scaffolds. Biochem. Biophys. Res. Commun..

[114] Born L.J., McLoughlin S.T., Dutta D., Mahadik B., Jia X., Fisher J.P., Jay S.M. (2022). Sustained released of bioactive mesenchymal stromal cell-derived extracellular vesicles from 3D-printed gelatin methacrylate hydrogels. J. Biomed. Mater. Res. A.

[115] Maiullari F., Chirivì M., Costantini M., Ferretti A.M., Recchia S., Maiullari S., Milan M., Presutti D., Pace V., Raspa M. (2021). In vivo organized neovascularization induced by 3D bioprinted endothelial-derived extracellular vesicles. Biofabrication.

[116] Nagappan P.G., Chen H., Wang D.Y. (2020). Neuroregeneration and plasticity: A review of the physiological mechanisms for achieving functional recovery postinjury. Mil. Med. Res..

[117] Liu X., Wang J., Wang P., Zhong L., Wang S., Feng Q., Wei X., Zhou L. (2022). Hypoxia-pretreated mesenchymal stem cell-derived exosomes-loaded low-temperature extrusion 3D-printed implants for neural regeneration after traumatic brain injury in canines. Front. Bioeng. Biotechnol..

[118] Liu Z., Tong H., Li J., Wang L., Fan X., Song H., Yang M., Wang H., Jiang X., Zhou X. (2022). Low-stiffness hydrogels promote peripheral nerve regeneration through the rapid release of exosomes. Front. Bioeng. Biotechnol..

[119] Yerneni S.S., Lathwal S., Shrestha P., Shirwan H., Matyjaszewski K., Weiss L., Yolcu E.S., Campbell P.G., Das S.R. (2019). Rapid on-demand extracellular vesicle augmentation with versatile oligonucleotide tethers. ACS Nano.

[120] Theodoraki M.-N., Yerneni S.S., Gooding W.E., Ohr J., Clump D.A., Bauman J.E., Ferris R.L., Whiteside T.L. (2019). Circulating exosomes measure responses to therapy in head and neck cancer patients treated with cetuximab, ipilimumab, and IMRT. Oncoimmunology.

[121] Jakab K., Neagu A., Mironov V., Markwald R.R., Forgacs G. (2004). Engineering biological structures of prescribed shape using self-assembling multicellular systems. Proc. Natl. Acad. Sci. USA.

[122] Mironov V., Visconti R.P., Kasyanov V., Forgacs G., Drake C.J., Markwald R.R. (2009). Organ printing: Tissue spheroids as building blocks. Biomaterials.

[123] Han J., Jeon S., Kim M.K., Jeong W., Yoo J.J., Kang H.W. (2022). In vitro breast cancer model with patient-specific morphological features for personalized medicine. Biofabrication.

[124] Kim M.K., Jeong W., Seunggyu Jeon S., Kang H.W. (2023). 3D bioprinting of dECM-incorporated hepatocyte spheroid for simultaneous promotion of cell-cell and –ECM interactions. Front. Bioeng. Biotechnol..

[125] Kang B., Park Y., Hwang D.G., Kim D., Yong U., Lim K.S., Jang J. (2022). Facile bioprinting process for fabricating size-controllable functional microtissues using light-activated decellularized extracellular matrix-based bioinks. Adv. Mater. Technol..

[126] Decarli M., Seijas-Gamardo A., Morgan F.L.C., Wieringa P., Baker M.B., Silva J.V.L., Moraes A.M., Moroni L., Mota C. (2023). Bioprinting of stem cell spheroids followed by post-printing chondrogenic differentiation for cartilage tissue engineering. Adv. Healthc. Mater..

[127] Kim M.H., Singh Y.P., Celik N., Yeo M., Rizk E., Hayes D.J., Ozbolat I.T. (2024). High-throughput bioprinting of spheroids for scalable tissue fabrication. bioRxiv.

[128] Pan B.T., Johnstone R.M. (1983). Fate of the transferrin receptor during maturation of sheep reticulocytes in vitro: Selective externalization of the receptor. Cell.

[129] Harding C., Heuser J., Stahl P. (1983). Receptor-mediated endocytosis of transferrin and recycling of the transferrin receptor in rat reticulocytes. J. Cell Biol..

[130] Pan B.T., Blostein R., Johnstone R.M. (1983). Loss of the transferrin receptor during the maturation of sheep reticulocytes in vitro. An immunological approach. Biochem. J..

[131] Valadi H., Ekström K., Bossios A., Sjöstrand M., Lee J.J., Lötvall J.O. (2007). Exosome mediated transfer of mRNAs and microRNAs is a novel mechanism of genetic exchange between cells. Nat. Cell Biol..

[132] Matai I., Kaur G., Seyedsalehi A., McClinton A., Laurencin C.T. (2020). Progress in 3D bioprinting technology for tissue/organ regenerative engineering. Biomaterials.

[133] Heinrich M.A., Liu W., Jimenez A., Yang J., Akpek A., Liu X., Pi Q., Mu X., Hu N., Schiffelers R.M. (2019). 3D bioprinting: From benches to translational applications. Small.

[134] Tavafoghi M., Darabi M.A., Mahmoodi M., Tutar R., Xu C., Mirjafari A., Billi F., Swieszkowski W., Nasrollahi F., Ahadian S. (2021). Multimaterial bioprinting and combination of processing techniques towards the fabrication of biomimetic tissues and organs. Biofabrication.

[135] Gu Z., Fu J., Lin H., He Y. (2020). Development of 3D bioprinting: From printing methods to biomedical applications. Asian J. Pharm. Sci..

[136] Gao B., Yang Q., Zhao X., Jin G., Ma Y., Xu F. (2016). 4D bioprinting for biomedical applications. Trends Biotechnol..

[137] Shimoda M., Khokha R. (2017). Metalloproteinases in extracellular vesicles. Biochim. Biophys. Acta Mol. Cell Res..

[138] Alqurashi H., Asencio I.O., Lambert D.W. (2021). The emerging potential of extracellular vesicles in cell-free tissue engineering and regenerative medicine. Tissue Eng. B.

[139] de Abreu R.C., Fernandes H., da Costa Martins P.A., Sahoo S., Emanueli C., Ferreira L. (2020). Native and bioengineered extracellular vesicles for cardiovascular therapeutics. Nat. Rev. Cardiol..

[140] Lai C.P., Mardini O., Ericsson M., Prabhakar S., Maguire C.A., Chen J.W., Tannous B.A., Breakefield X.O. (2014). Dynamic biodistribution of extracellular vesicles in vivo using a multimodal imaging reporter. ACS Nano.

[141] Piffoux M., Silva A.K.A., Wilhelm C., Gazeau F., Tareste D. (2018). Modification of extracellular vesicles by fusion with liposomes for the design of personalized biogenic drug delivery systems. ACS Nano.

[142] U shell effective Decarli M.C., Mizukami A., Azoubel R.A., Neto P.I., Mota C., Moraes A.M., Silva J.C.L., Moroni L. (2022). Static systems to obtain 3D spheroid cell models: A cost analysis comparing the implementation of four types of microwell array inserts. Biochem. Eng. J..

[143] Zhang Y., Liu Y., Liu H., Tang W.H. (2019). Exosomes: Biogenesis, biologic function and clinical potential. Cell Biosci..

[144] Sreeraj H., AnuKiruthika R., Tamilselvi K.S., Subha D. (2024). Exosomes for skin treatment: Therapeutic and cosmetic applications. Nano TransMed.

[145] Thakur A., Shah D., Rai D., Parra D.C., Pathikonda S., Kurilova S., Cili A. (2023). Therapeutic values of exosomes in cosmetics, skin care, tissue regeneration, and dermatological diseases. Cosmetics.

[146] Bai G., Truong T.M., Pathak G.N., Benoit L., Rao B. (2024). Clinical applications of exosomes in cosmetic dermatology. Skin Health Dis..

[147] Wang Q., Liu Y., Zhang S., He F., Shi T., Li J., Wang Z., Jia J. (2023). Exosome-based bioinks for 3D bioprinting applications in tissue engineering and regenerative medicine. Int. J. Bioprinting.

